# Roluperidone monotherapy, a treatment for negative symptoms in schizophrenia

**DOI:** 10.1093/ijnp/pyaf032

**Published:** 2025-05-20

**Authors:** Michael Davidson, Nadine Noel, Florent Schmitt, Remy Luthringer

**Affiliations:** Minerva Neurosciences, 1500 District Avenue Burlington, MA 01803, USA; Clinical and Basic Sciences Department Nicosia University Medical School, Nicholas St 93, Egkomi Lefkosias 2408, Cyprus; PPRS SAS, PPRS, 4e Av. du Général de Gaulle, 68000 Colmar, France; PPRS SAS, PPRS, 4e Av. du Général de Gaulle, 68000 Colmar, France; Minerva Neurosciences, 1500 District Avenue Burlington, MA 01803, USA

**Keywords:** schizophrenia, negative symptoms, treatment, roluperidone

## Abstract

Roluperidone is a drug in development targeting primary negative symptoms in schizophrenia, which binds sigma-2, 5-HT_2A,_ and alpha_1a_ receptors. Roluperidone administered as monotherapy to patients suffering from mild to moderate negative symptoms and withdrawal from antipsychotic drugs, improved negative symptoms in 2 clinical trials. The patients who were symptomatically stable for 3 or 6 months before antipsychotic drug withdrawal showed a very low rate of psychotic symptoms worsening over 6 or 9-month trial duration.

Focus: Treatment of primary negative symptoms in a subgroup of patients suffering from schizophrenia.

## Background

Schizophrenia is characterized by intermittent psychosis, negative symptoms, and mild to moderate cognitive impairment. Drugs which ameliorated acute psychosis and reduce the risk for future psychotic exacerbation have been available since the early 60s and are widely used in clinical practice. Recently, Xanomeline, a muscarinic agonist with no direct blocking effect on dopamine receptors, has been added to the drugs indicated for the treatment of schizophrenia. However, there currently exist no drugs which ameliorate negative symptoms or cognitive impairment. While the main reason for this deficiency is the poor understanding of schizophrenia pathophysiology, it is possible that the adherence to the Diagnostic and Statistical Manual/International Classification of Disease (DSM/ICD) constructs in drug development has been an obstacle rather than a facilitator. DSM schizophrenia is a phenomenological construct of convenience which encompasses individuals who differ widely in terms of illness manifestation, course, response to treatment, and most probably biological abnormalities. Nevertheless, administrative and practical reasons impose that the boundaries of schizophrenia be defined within agreed upon diagnostic systems such as DSM and ICD. In clinical practice, individuals meeting DSM criteria for schizophrenia were supposed to be matched to appropriate treatments and in research to be the focus of pathohistological and etiologic research. Unfortunately, the current diagnostic systems have contributed only partially to the match between individual patients and available treatments for schizophrenia. On one hand, about 1/3 of the patients who meet criteria for schizophrenia do not benefit at all, and many benefit only temporarily from available treatments,^1^ not to mention those who abandon antipsychotic treatment on their own because of poor tolerability. On the other hand, antipsychotic treatment is not specific to schizophrenia; hence, the match between treatment and diagnostic classification has not been very successful. Genetic studies have posed the strongest challenge to the biological validity of DSM/ICD schizophrenia as a discrete entity.^2^ The average genetic correlation is > 0.20 between schizophrenia, bipolar disorder, major depressive disorder, attention deficit hyperactivity disorder (ADHD), anorexia nervosa, obsessive–compulsive disorder, autism spectrum disorder, and Tourette syndrome, and 0.70 between schizophrenia and manic-depressive illness.^3,4^ Likewise, occasional mean group differences in biological markers distinguishing between schizophrenia patients and controls in imaging and neurochemical studies are either driven by a few outliers or not replicated. The leading unitary hypothesis for schizophrenia, the hyperdopaminergic/ dysregulation Dopamine (DA) hypothesis, which was the subject of over 40 years of research,^5^ is about to be abandoned or radically modified.

Supporting the assertion of schizophrenia heterogeneity would be the following example, highlighting the implausibility that the 2 individuals described below —both meeting DSM criteria for schizophrenia—share the same pathohistological abnormality: one a married mother of 2 with an Intelligence Quotient (IQ) of 105, persistent auditory hallucinations, paranoid delusions, no negative symptoms, holding a steady paying job and another male individual with an IQ of 85, severe thought disorder, mild and intermittent delusions, and severe and persistent negative symptoms who never married or had a job. If one accepts the biological heterogeneity notion, always treating all patients with the same drugs that interfere with DA neurotransmission is an exercise in futility. Delusions, hallucinations, and thought disorders are essential symptoms for the diagnosis of schizophrenia, and negative symptoms and cognitive impairment are very frequent but not essential. Ideally, a single drug treatment should prevent all the emotional and behavioral manifestations grouped under the umbrella of schizophrenia, the way penicillin could prevent or treat the emotional and behavioral manifestation of spirochaete.^6^ However, the more likely scenario is that as the specific symptoms of schizophrenia and their respective interacting pathophysiological processes are better understood and dissected into the most basic manifestations such as auditory hallucinations, delusions, avolition, apathy, each symptom will be treated as it manifests with specific drugs targeting the specific manifestation.^7^ This might be true even if it turns out that some schizophrenia symptoms derive from common pathobiological or etiologic backgrounds. Chest pain, cardiac arrhythmia, dyspnea, and peripheral edema can all be tracked to a common coronary pathophysiology on the background of a common metabolic abnormality, yet each symptom is treated with different drugs as it manifests.

The general idea for revising the relationship between psychiatric syndromes and drug development is to (1) identify basic symptoms that manifest transdiagnostically across DSM syndromes, (2) identify biological circuits or genetic-molecular processes and markers common to these manifestations, and (3) devise therapeutic interventions that engage targets common to these circuits and/or genetic-molecular processes. Research Domains Criteria^8^ and Psychiatric Ratings using Intermediate Stratified Markers^9^ are diagnostic systems attempting to address this issue.

Negative symptoms are multidimensional conditions manifesting in about 2/3 of individuals affected by schizophrenia. Avolition, apathy, anhedonia, asociality, blunted affect, and alogia classified under the umbrella of negative symptoms manifest in several DSM diagnostic categories and occasionally in individuals who do not fit in any specific DSM diagnostic category.^10^ Depending on the specific DSM category, specific manifestations of negative symptoms can prevail. For example, avolition may be more prominent in schizophrenia, apathy is more prominent in Parkinson’s and Alzheimer’s disease, anhedonia in major depression, and alogia and asociality in autism.^10,11^

Because of their large prevalence and undesirable impact on quality of life, almost every compound presumed to penetrate into the Central Nervous System (CNS) has been tried as a treatment in negative symptoms. Antidepressants, stimulants like amphetamine and ritalin, modafinil, compounds affecting N-methyl-D-aspartate (NMDA) receptors like glycine derivatives, pharmacological manipulations of the trace amine-associated receptor 1, PDE10A inhibition cannabinoids, and psychedelics are an incomplete list of such attempts to treat negative sympotms.^12,13^

Antipsychotic drugs alone or in combination with another compound have been the most frequently tried approach to treat negative symptoms. Indeed, antipsychotic drugs can improve social interaction by ameliorating frightening delusions and hallucinations but might also increase avolition/anhedonia by interfering with the brain’s DA-driven reward system. In addition, most antipsychotics induce sedation, Parkinsonism, and akathisia, which in turn induce secondary negative symptoms in addition to the schizophrenia intrinsic primary negative symptoms. Therefore, a group of schizophrenia experts led by Steven Marder and sponsored by the International Society for CNS Trials proposed an alternative design by which drug studies targeting negative symptoms should administer the experimental drug as monotherapy and compare it to placebo. This approach allows to investigate the direct therapeutic effect of the experimental drug on primary negative symptoms without the confounding impact of the secondary negative symptoms produced by antipsychotic drugs. Once an effective drug for negative symptoms is developed to apply the monotherapy approach in clinical practice, it is necessary to identify these schizophrenia patients who can maintain stability of psychotic symptoms in the absence of antipsychotic medication. While the search for reliable biological markers predicting stable remission is still in course, there exist several phenomenological manifestations which clinicians and researchers employ to identify this subgroup of patients. These manifestations are general stability of all symptoms, in particular those related to agitation and violence, no need for treatment change or hospitalization, moderate to high negative symptoms, all this for at least 3 to 6 months.^14^

Roluperidone, Preclinical, Roluperidone(1H-Isoindol-1-one,2-[[1-[2-(4-fluorophenyl)-2- oxoethyl]-4-piperidinyl]methyl]-2,3-dihydro-, hydrochloride, hydrate (1:1:2)) is a novel cyclic amido derivative. As determined by Alon et al^15^ and summarized by Ye et al,^16^ the co-crystal structure with the sigma-2 receptor reveals that the protonated nitrogen atom in the piperidine ring forms a typical salt bridge with the conserved Asp29, which subsequently creates a hydrogen bond with another conserved acidic amino acid residue, Glu73, located 3 Å away. Additionally, the fluorobenzene fragment extends into a deep hydrophobic pocket within the receptor, and this fluorine atom establishes a crucial halogen bond with Glu77.

In in vitro receptor binding studies, roluperidone acts as an antagonist with high affinity to sigma-_2_, 5-HT_2a_, and alpha_1a_-adrenergic receptors (K_i_ = 7.53, 8.19, 4.17 nmol/L, respectively) and weak affinities to sigma-_1._ The affinity for DA receptors (D1 to D5) is weak (median inhibitory concentration, IC_50_ > 1000 nmol/L).

The functional effect of roluperidone at sigma-_2_ receptors was tested using the selective model of injection of 1,3-Di-o-tolylguanidine (DTG) into the red nucleus (RN) to induce neck dystonia in rats.^17,18^ Roluperidone significantly prevented DTG-induced neck dystonia at 1 and 3 mg/kg, while when injected into the RN alone, it did not produce any postural change. Thus, this unique functional assay confirms that roluperidone is an antagonist at sigma-_2_ receptor.

Using the head-twitch response induced by 5-hydroxytryptophan, the anhydrous form of roluperidone, orally administered to male Wistar rats at the dose of 0.27mg/kg, significantly reduced the number of head-twitches, suggesting an antagonistic activity at 5-HT_2A_ receptors.

To determine the binding properties of roluperidone on the alpha_1A_ adrenergic receptor, the receptor activation (intracellular Ca2 + concentration) was assessed in human recombinant CHO cells. Roluperidone demonstrated antagonist activity at the alpha-_1A_ adrenergic receptor. The potential of roluperidone to affect symptoms of schizophrenia was tested in several behavioral paradigms in Wistar rats. The repeated administration of PCP NMDA receptor antagonist phencyclidine [PCP] is considered to have translational relevance to the negative symptoms of schizophrenia.^19-21^ Oral repeated administration during 10 days of 1 and 3 mg/kg of roluperidone in rats significantly inhibited PCP-induced decrease in social interaction time, suggesting a potential effect of roluperidone on negative symptoms of schizophrenia. Roluperidone also tested positive in rat behavioral models predictive of positive symptoms improvements such as reducing locomotor activity in response to methamphetamine or apomorphine or noncompetitive NMDA glutamate receptor antagonists.

In vitro roluperidone increased brain-derived neurotrophic factor (BDNF) release by astrocytes and hippocampal neurons, an effect which might be modulated by alpha-_1A_ adrenergic and sigma receptors, known to influence astrocyte activation via Ca^2+^ signals. Reduced expression of BDNF has been identified in the frontal cortex and hippocampus in patients with schizophrenia,^22^ and studies have found that BDNF may play a role in the neuropsychological functions of schizophrenic.^23^

Although the mechanism of action of roluperidone is not completely understood, the overall results obtained, and its unique and specific pharmacological profile suggest a complex mechanism of action that may affect the neurotransmitter pathways involved in the regulation of negative symptoms of schizophrenia. Additionally, this investigational compound may have the potential for disease modification and improved neuroplasticity.

### Roluperidone Clinical

After single-dose administration of 64 mg GR01/C to healthy subjects, roluperidone was absorbed with a median Tmax of 3.5 hr, a Cmax of 32.1 ng/mL, an AUCinf of 312 ng·hr/mL, and a half-life of about 7 hrs. The fraction absorbed ranged from 73% to 81%. Typical values for CL/F and V/F were estimated at 152 L/hr and 1500 L, respectively. Pharmacokinetics (PK) parameters observed in patient populations were the same as for healthy subjects. The presence of food slowed the rate of absorption of roluperidone, but no significant differences in exposure were observed between the fed and fasted state. No clinically relevant differences between male and female subjects were observed in the PK of roluperidone.

A phase 2 and a phase 3 clinical trial which together enrolled 775 patients were conducted. The recruited schizophrenia patients had stable symptoms for 3^24^ or 6 months,^25^ had scores > 20 on the PANSS negative symptoms subscale and scores ≤ 4 on items reflective of agitation (P4 Excitement, P6 suspiciousness/persecution, P7 hostility, G8 uncooperativeness, and G14 poor impulse control). The main outcome measure was negative symptoms constructs derived from the Positive and Negative Symptoms Scale (PANSS) and the main secondary outcome was the Personal Social Performance. Patients were treated with roluperidone 32 mg, 64 mg, or placebo for 12 weeks, at which time they could continue on the same dose of roluperidone open label for 6 months in the phase 2^24^ trial and for 9 months in the phase 3 trial.^25^

Results of the 2 trials demonstrated that roluperidone 64 mg/day administered as *monotherapy* separated from placebo at statistical significant level (< .005) for negative symptoms and social functioning.^24,25^ A network analysis indicates that avolition might be the “driver” of the drug effect on negative symptoms.^26^.The BACS token motor, verbal fluency, and composite z scores showed significant improvements in the 32 mg group compared to the placebo group with some correlations between negative symptoms and aspects of cognitive performance.^27^ During the open label 6 or 9 months of roluperidone treatment, negative symptoms and social functioning continue to improve^14^ and the rate of psychotic exacerbation or hospitalization was below 12% which is definitely not higher that what could be expected in patients under maintenance treatment with antipsychotics.^14^ During the open-label phase, investigators discontinued roluperidone and administered an antipsychotic drug in 9.1% of the patients in study 1 and in 8% of the patients in study 2 consistent with the rate of exacerbation in the Randomized Controlled Trial (RCT) phases of the studies.^14^ It is possible that the 5-HT_2_ receptor blocking property, which is common to roluperidone and most antipsychotics, is responsible for the low rate of psychotic exacerbation, consistent with studies reported in the literature.^28^ The trials described were approved by the respective IRBs and were conducted according to the local national regulations.

In summary, roluperidone, which is currently under development, could be an effective drug for a subgroup of schizophrenia patients whose psychotic symptoms have been stable over 3 to 6 months, but negative symptoms persist and have a harmful impact on their quality of life and potential on other individuals in whom avolition/ apathy manifests.

## Data Availability

Data supporting the findings of this study are available from the corresponding author upon reasonable request.
